# Recursive module extraction using Louvain and PageRank

**DOI:** 10.12688/f1000research.15845.1

**Published:** 2018-08-14

**Authors:** Dimitri Perrin, Guido Zuccon

**Affiliations:** 1 School of Electrical Engineering and Computer Science, Queensland University of Technology, Brisbane, QLD, 4001, Australia

**Keywords:** Network biology, Module identification, Community detection, DREAM challenge

## Abstract

Biological networks are highly modular and contain a large number of clusters, which are often associated with a specific biological function or disease. Identifying these clusters, or modules, is therefore valuable, but it is not trivial. In this article we propose a recursive method based on the Louvain algorithm for community detection and the PageRank algorithm for authoritativeness weighting in networks. PageRank is used to initialise the weights of nodes in the biological network; the Louvain algorithm with the Newman-Girvan criterion for modularity is then applied to the network to identify modules. Any identified module with more than *k* nodes is further processed by recursively applying PageRank and Louvain, until no module contains more than *k* nodes (where *k* is a parameter of the method, no greater than 100). This method is evaluated on a heterogeneous set of six biological networks from the Disease Module Identification DREAM Challenge. Empirical findings suggest that the method is effective in identifying a large number of significant modules, although with substantial variability across restarts of the method.

## Introduction

Biological functions emerge from interactions at the molecular level. For instance our circadian clock relies on the interactions between a large number of genes and proteins ^1, 2^, and many cancer types are typically associated with specific genetic ^3^ and epigenetic ^4^ modifications. Unsurprisingly, biological networks such as protein-protein interaction (PPI) or regulatory networks therefore have a high degree of modularity (a measure of strength of the division of the network into subgroups, or clusters, called *modules* in our context) where the ‘modules’ often correspond to genes or proteins that are involved in the same biological functions. Diseases are also rarely associated with a single gene: disease genes have a high propensity to interact with each other, forming disease modules ^5^. The identification of these disease modules is a valuable tool to identify disease pathways, but also to predict other disease genes.

This task is sometimes also known as community detection or graph clustering. This is a well established problem in network science. A large number of methods exist (see e.g., 6), but there was a lack of common evaluation on relevant biological networks.

The Disease Module Identification DREAM Challenge aimed to comprehensively assess module identification methods across six diverse, unpublished molecular networks ^7^. Participating teams were tasked to predict disease-relevant modules both within individual networks (subchallenge 1) and across multiple, layered networks (subchallenge 2). The modules were defined as non-overlapping subsets containing 3 to 100 nodes. This is not a graph partition task, as not all nodes necessarily have to be assigned to a module.

In this article, we detail our solution for subchallenge 1. Next, we introduce the six networks and how we preprocessed them, then we describe our recursive algorithm, and discuss its performance across each network.

## Methods

### Networks

The human molecular networks used in the challenge are described in the challenge overview paper ^7^. For convenience, we summarise their main characteristics in Table 1. On top of capturing different types of biological information, they also vary in terms of size, link density and structural properties.

**Table 1.** Challenge networks.

ID	Type	# nodes	# edges	Directed
1	PPI	17,397	2,232,405	No
2	PPI	12,420	397,309	No
3	Signalling	5,254	21,826	Yes
4	Co-expression	12,588	1,000,000	No
5	Cancer	14,679	1,000,000	No
6	Homology	10,405	4,223,606	No

For the duration of the challenge, networks were only provided in anonymised form, without any gene names, details on the underlying data or how the networks were constructed. In the experiment in this article, we also considered networks in their anonymised form.

While protein interaction and homology networks, for instance, are obviously very different in nature, we opted to develop a method that could be applied to any network, independently of its type (although some preprocessing, described next, may be required, along with network-specific parameter tuning). This was because of the constraints of the challenge, in terms of both time and limited number of submissions.

### Pre-processing

To have a method that works across network types, we decided to focus only on undirected networks. We also assumed that edge weights are in the range [0, 1]. Most networks in the challenge satisfy these requirements; pre-processing was applied to the remaining networks.

**Figure 1.**
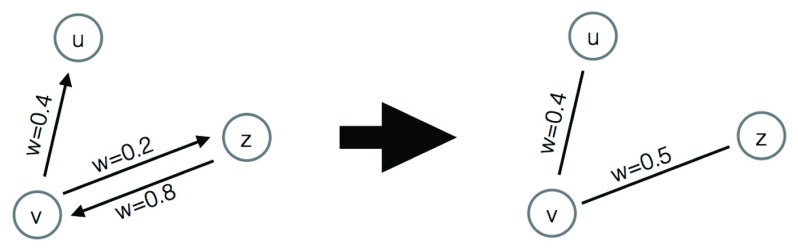
Conversion of a directed network into an undirected one.

Network 3 is a directed network and as such needed to be converted to an undirected representation. This was achieved by simply assigning to all undirected < *u*, *v*> edges the average of the weights of the directed ( *u*, *v*) and ( *v*, *u*) edges (see Figure 1).

Networks 3 and 6 required normalisation of their weights. This was achieved by dividing all the original weights in each network by the maximum weight in that network.

These standardised networks are used as an input to our method. In what follows, any mention of a network refers to its standardised version.

### Algorithm

The core of our method is the greedy Louvain algorithm ^8^. This algorithm is a well-established method for community detection in networks ^6^, it is applicable to weighted networks, and it provides better modularity maxima than other available greedy techniques ^6^. In addition, the algorithm is computationally efficient and even large networks can be analyzed in reasonable runtime.

The algorithm starts by creating communities of size 1 where each node in the network forms a community. Then the algorithm proceeds by executing two steps. In the first step, the algorithm attempts to assign a node *v* to a community of a neighbor *u*, such that the modularity of the partition is increased. This process is repeated for as long as the modularity can be improved. This process generates an initial partition of the network. In the second step of the algorithm, each community of the partition is treated as a supernode. Supernodes are connected if at least one edge exists between nodes of each community the supernodes represents. Once this second step is concluded, the algorithm iterates and stops when the modularity cannot increase anymore.

As part of our methods, we rely on the implementation of Louvain (v0.2) by Blondel *et al.* ^8^. The Louvain algorithm does not explicitly identify which modularity criterion is required: indeed, the algorithm can be instantiated using a number of modularity criteria. Their implementation supports ten modularity criteria; in all our submissions we used the default Newman-Girvan criterion ^9^.

By default, in the Louvain algorithm, the initial partition assigns each node to a module that contains only the node itself. This creates a lot of variability in the results, which we reduced by modifying the algorithm. An idealised module is similar to a clique: it would contain nodes that are highly connected to other nodes, which are highly connected to similar nodes, etc. In other words, a node is important if it is linked to other nodes that are important. This closely matches the intuition of the PageRank algorithm developed to score web pages ^10^. PageRank has been widely used in settings other than web search, including in bioinformatics ^11^. Our solution is therefore to calculate the PageRank for each node of the network, and to create an initial partition where each node is allocated to the module corresponding to its highest-scored neighbor (or itself, if that neighbor is scored lower). This has the advantage of both reducing the variability and ‘seeding’ Louvain with a promising partition. Here, we used a modified PageRank score that takes into account the edge weights.

Given that the task was to find modules with 3 to 100 nodes, a simple approach could be to run Louvain, process layer 1 from the hierarchical output generated by the algorithm, and extract all modules with a suitable size. This is, of course, far from optimal: Louvain generates modules of any size, and there may be interesting modules ‘hiding’ in a module containing more than 100 nodes (which would not be a valid submission to the challenge).

Initial tests on trimming or splitting large modules did not yield any useful results, so we implemented a recursive approach. For any network of size greater than *k* (for instance, *k* = 100), we run Louvain and process all modules. If a module contains between 3 and *k* nodes, it is saved. If it contains less than 3 nodes, it is discarded. If it contains more than *k* nodes, we extract the corresponding network and add it to a list of networks to which Louvain is recursively applied. The recursion terminates when this list is empty. PageRank-based initialisation is used for all recursion levels.

**Figure 2.**
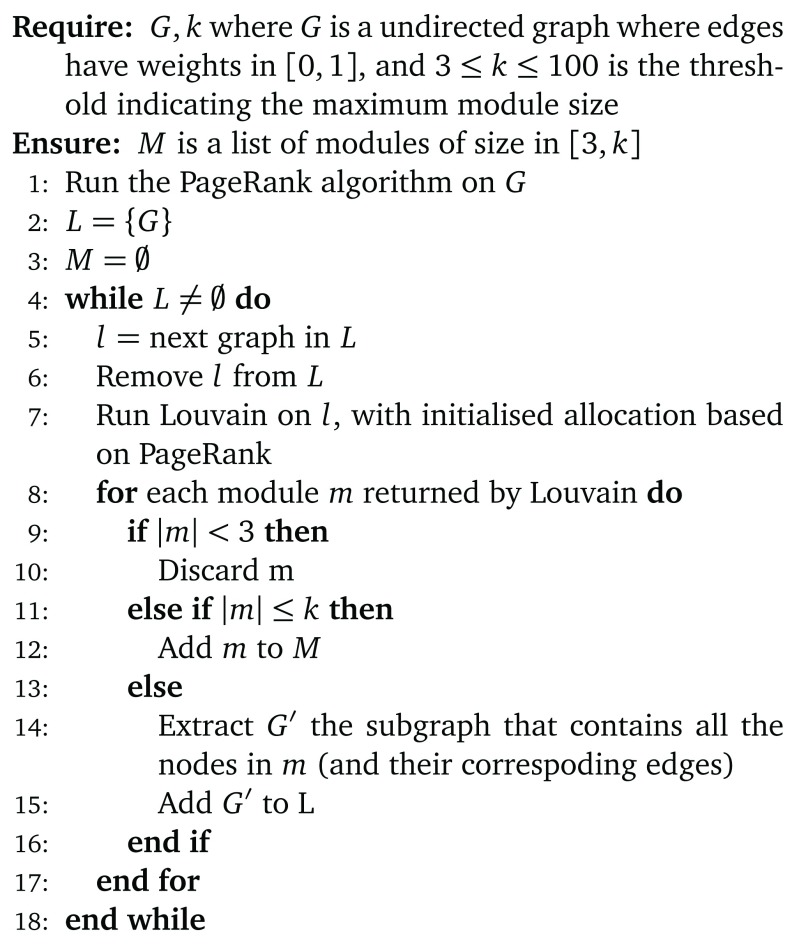
Overall algorithm.

The overall algorithm is summarised in Figure 2.

### Evaluation

During the challenge, modules extracted from the anonymised networks were submitted to the online platform and evaluated by the organisers. Modules were scored using the Pascal tool for pathway scoring ^12^. For each submission, the organisers would then communicate the number of significant modules that were identified for each of the six networks, but without providing any information on which submitted modules were significant. In the challenge leaderboard, submissions were ranked by the total number of significant modules identified. In this article, we analyse additional runs of our algorithm, evaluated locally using the code and GWAS data released by the organisers. Running the evaluation locally allows us to know which modules are significant.

The two parameters of our algorithm are the network being processed, and the value of the threshold *k* for the recursion. One configuration is a pair of a network and a threshold. For each configuration, we performed 10 runs of our algorithm.

## Results

On the final challenge leaderboard, our solution ranked 12 ^th^ overall with 44 significant modules identified across the six networks (when the winning team found 60). Relative to other teams, it performed best on network 2 (10 modules found, best score 13) and network 3 (7 modules found, best score 9).

**Figure 3.**
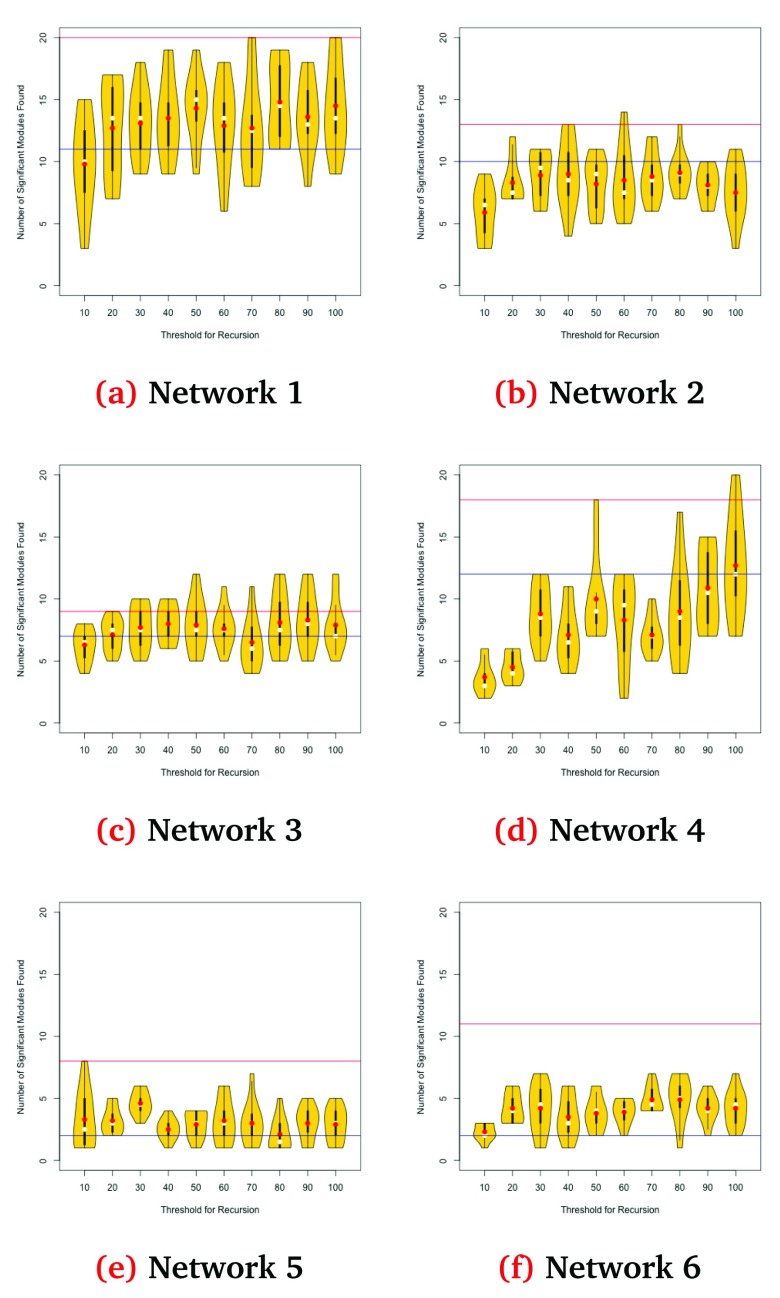
Results on each network as a function of the value for *k*. White and red dots represent the median and mean values for each configuration, respectively. The blue line indicates our performance in the challenge leaderboard for that network, and the red line that of the best submission for that network.

Here, we analyse the performance over 100 new runs (10 per threshold value) for each network. The results are shown in Figure 3.

Louvain is non-deterministic, and even after initialising it using PageRank, the results for any given configuration have high variability. It is also worth noting that, for five of the six networks, there is at least one configuration for which our algorithm matches or outperforms the best system submitted to the challenge. Only network 6 leads to poor results. If we combine the best result for each network, we obtain a theoretical total of 81 significant modules, close to double our final score and 35% better than the best-performing solution in the challenge.

For most networks the performance is robust to changes of *k*, but there still appears to be an optimal configuration for each network. For networks 1, 3 and 4, our method produces better results with large values of *k*. For network 5, aiming for smaller modules produced better results, while for network 2 mid-range values of *k* are preferable.

## Discussion

The results from these 600 additional runs show the potential of our approach. Under the same conditions as the challenge, our algorithm can match or improve the best results from the competition phase.

Evaluating all the modules from a given solution against all the GWAS data using Pascal takes hours, and it is therefore not practical to use this evaluation to guide the creation of the modules. Even outside the challenge, it is more realistic for the extraction method to be purely driven by the network itself.

However, now that the challenge is completed, it is possible to evaluate thousands of modules. Using this data, future work will focus on developing a module ‘score’ that would be a good predictor of whether that module is significant. If this can be achieved, we would then add a local optimisation step at the end of our algorithm, to fine tune the extracted modules.

Another direction for future work is to study the consensus between restarts. How many times do we identify the same modules, or does this correlate with their significance? We believe there is potential for voting/fusion approaches to extend our algorithm.

## Conclusions

Network-based approaches are an important tool in biomedical research, as they can lead to the identification of clusters of genes (modules) involved in the same molecular function or the same disease.

Identifying these modules is not trivial, and the Disease Module Identification DREAM Challenge was an important initiative to benchmark various approaches. We developed a recursive method based on the Louvain and PageRank algorithms, which performed reasonably well in the challenge.

Here, we showed that this method can actually match or exceed the best results from the competition challenge. Further work will focus on exploiting the high variability between restarts, and on developing a module score that can guide optimisation of the identified modules.

## Data availability

The dataset associated with the Disease Module Identification DREAM Challenge is available for registered participants at http://www.synapse.org/#!Synapse:syn6156761/wiki/400659.

Challenge results and scoring scripts are available at http://www.synapse.org/#!Synapse:syn6156761/wiki/400647.

## Software availability

Source code implementation for the recursive method presented in this article and used in the Disease Module Identification DREAM Challenge is available from GitHub: https://github.com/bmds-lab/DMI/tree/v0.1

Archived source code at time of publication https://doi.org/10.5281/zenodo.1330835 ^13^.

Source code is available under a GLP 3.0 license
