# Supplementary material for: Recursive module extraction using Louvain and PageRank
Source: F1000Res. 2018 Aug 14;7:1286. [Version 1] doi: 10.12688/f1000research.15845.1 (PMC6143918; doi:10.12688/f1000research.15845.1)
Supplement: Peer review history

# Peer review: Referee response for version 1

**DOI:** 10.5256/f1000research.17297.r37207

**Published:** 2018-09-14

**Authors:** Alina Sîrbu

**Affiliations:** 1 Department of Computer Science, University of Pisa, Pisa, Italy

The paper presents a recursive algorithm to find modules in biological networks. The authors evaluate the modules using a DREAM dataset. The algorithm is promising and the paper is well written. The results are comparable and sometimes better than the best performance of the DREAM Challenge. I think some changes could improve the paper:

 1. In the definition of the algorithm, the intuition between the concept of modularity could be included, and maybe a high level definition of  the chosen modularity index.

 2. “By default, in the Louvain algorithm, the initial partition assigns each node to a module that contains only the node itself. This creates a lot of variability in the results” Do you mean variability from one run to another?

 3. “For each configuration, we performed 10 runs of our algorithm.”  Here you are evaluating each run and showing all 10 results. I wonder what would happen if one combined modules from different runs. To create an ‘ensemble’ module extractor. One could just pool together the modules found. This could be done over the 10 runs with the same K, but also maybe over runs with different k? Or even changing the modularity criterion and combining the results. And then selecting the most frequently found modules...

 4. For figure 3: is there information on how many significant modules are actually known in the networks? One could think of adding another line that would show the number of significant modules known as an upper bound for the performance.

 5.  “If we combine the best result for each network, we obtain a theoretical total of 81 significant modules, close to double our final score and 35% better than the best-performing solution in the challenge”.  How is this combination made?

I have read this submission. I believe that I have an appropriate level of expertise to confirm that it is of an acceptable scientific standard.

# Peer review: Referee response for version 1

**DOI:** 10.5256/f1000research.17297.r37208

**Published:** 2018-09-10

**Authors:** Raghvendra Mall

**Affiliations:** 1 Qatar Computing Research Institute (QCRI), Hamad Bin Khalifa University (HBKU), Doha, Qatar

The article proposes usage of a recursive form of Louvain method while including the PageRank of nodes to make graph partitions or detect biological 'modules' which are then evaluated through the DREAM Challenge evaluation tool PASCAL to determine how many of the identified modules were significant w.r.t. GWAS experiments  conducted by the DREAM Challenge organisers.

 The paper has been well written and all the technical details have been elaborated quite well by the authors, thereby suggesting that the method is reproducible and can be extended as per the suggestion of the authors to have a consensus disease module identification technique. 

 The authors provide a good introduction to Louvain method explaining its non-deterministic nature and limitations such as resolution limit for which it needs to be used in a recursive fashion to detection modules of length k ([3,100]). Moreover, they explain well how PageRank is used along with the Louvain method.

 The only issue that I have is with the experiment section where the authors perform an additional 100 new runs and claim that they can obtain theoretically 81 significant modules. This is not correct way of evaluation as the authors are using the test set and tuning their hyper-parameters on the test set. In order to have a generic model, the authors can tune their model parameters on the training set and use the same for each test set network rather than tuning the results on test set. The authors do indicate this when they say that in future work they will focus on developing a module 'score' to predict if a module is significant or not. 

 A major issue here is the non-deterministic nature of Louvain method which will result in different partitions every time the code is run. Hence the idea of having a 'consensus between restarts' is also interesting. 

 Finally, it would have been better if the authors add information about the biological content of the modules that they have discovered and for which GWAS traits were the modules enriched in a given population. That analysis would complete the paper from a biological standpoint also.

I have read this submission. I believe that I have an appropriate level of expertise to confirm that it is of an acceptable scientific standard.
